# *Blastocystis* sp. Carriage and Irritable Bowel Syndrome: Is the Association Already Established?

**DOI:** 10.3390/biology10040340

**Published:** 2021-04-19

**Authors:** Fernando Salvador, Beatriz Lobo, Lidia Goterris, Carmen Alonso-Cotoner, Javier Santos, Elena Sulleiro, Begoña Bailo, David Carmena, Adrián Sánchez-Montalvá, Pau Bosch-Nicolau, Juan Espinosa-Pereiro, Isabel Fuentes, Israel Molina

**Affiliations:** 1 Department of Infectious Diseases, Vall d’Hebron University Hospital, PROSICS Barcelona, Pº Vall d’Hebron 119-129, 08035 Barcelona, Spain; adsanche@vhebron.net (A.S.-M.); pau.boschnicolau@gmail.com (P.B.-N.); macspinosa@gmail.com (J.E.-P.); imolina@vhebron.net (I.M.); 2 Digestive System Research Unit, University Hospital Vall d’Hebron, Centro de Investigación Biomédica en Red de Enfermedades Hepáticas y Digestivas (Ciberehd); Departament de Medicina, Universitat Autònoma de Barcelona, 08193 Cerdanyola del Vallès, Spain; bealoboster@gmail.com (B.L.); 33925mac@gmail.com (C.A.-C.); santosjav@gmail.com (J.S.); 3 Department of Microbiology, Vall d’Hebron University Hospital, PROSICS Barcelona, 08035 Barcelona, Spain; lgoterris@vhebron.net (L.G.); esulleir@vhebron.net (E.S.); 4 National Centre of Microbiology, Instituto de Salud Carlos III, 28220 Madrid, Spain; BEGOBB@isciii.es (B.B.); dacarmena@isciii.es (D.C.); ifuentes@isciii.es (I.F.)

**Keywords:** *Blastocystis* sp., irritable bowel syndrome, pathogenesis

## Abstract

**Simple Summary:**

The intestinal protist *Blastocystis* sp. is one of the most common intestinal parasites observed in humans, and has a worldwide distribution, being more prevalent in developing countries. Although this parasite has been described decades ago, the pathogenic potential it is still not understood completely. It has been suggested that *Blastocystis* sp. could be related with irritable bowel syndrome, a functional gastrointestinal disorder characterized by abdominal pain, discomfort with defecation, and changes in the frequency or form of stool. In our study, we compare a group of patients with irritable bowel syndrome with a group of healthy volunteers; no differences regarding the occurrence of *Blastocystis* sp. detection was found between both groups.

**Abstract:**

Background: The aim of the present study is to describe the occurrence of *Blastocystis* sp. detection among asymptomatic subjects and patients with irritable bowel syndrome in order to evaluate the potential association between irritable bowel syndrome and the parasitic infection. Methods: Cross-sectional study where adult patients with irritable bowel syndrome diagnosed according to Rome IV criteria were included. A control group was formed by asymptomatic subjects older than 18 years. Exclusion criteria were: immunosuppressive condition or having received any drug with demonstrated activity against *Blastocystis* sp. within the last 6 months before study inclusion. Epidemiological and clinical information was collected from all included participants. Two stool samples were obtained from all participants: one sample for microscopic examination and one sample for *Blastocystis* sp. PCR detection. *Blastocystis* sp. infection was defined by the positivity of any of the diagnostic techniques. Results: Seventy-two participants were included (36 asymptomatic subjects and 36 patients with irritable bowel syndrome). Thirty-five (48.6%) were men, and median age of participants was 34 (IQR 29–49) years. The overall rate of *Blastocystis* sp. carriage was 27.8% (20/72). The prevalence assessed through microscopic examination was 22.2% (16/72), while the prevalence measured by PCR was 15.3% (11/72). When comparing the presence of *Blastocystis* sp. between asymptomatic subjects and IBS patients, we did not find any statistically significant difference (36.1% vs. 19.4% respectively, *p* = 0.114). Conclusions: regarding the occurrence of *Blastocystis* sp., no differences were found between asymptomatic participants and patients with irritable bowel disease irrespective of the diagnostic technique performed.

## 1. Introduction

The protist *Blastocystis* sp. (superphylum Heterokonta) is one of the most common intestinal parasites observed in humans, with up to one billion estimated humans colonized. *Blastocystis* sp. has a worldwide distribution, being more prevalent in developing countries [1]. This parasite is transmitted by the fecal-oral route, and transmission can occur from human to human or from animal to human [2]. Studies based on the comparison of the nuclear small subunit rRNA gene show that *Blastocystis* sp. has an extensive molecular diversity; twenty-three subtypes (STs) have been described, although only STs 1–9 and ST12 have been found in humans [3,4].

The pathogenic potential of *Blastocystis* sp. is a matter of debate since it has been detected in both asymptomatic and symptomatic individuals. When present, clinical features associated with *Blastocystis* sp. include gastrointestinal symptoms (diarrhea, abdominal pain, vomiting, flatulence, bloating, dyspepsia, and other gastrointestinal symptoms), and cutaneous manifestations such as chronic urticaria [5,6]. Different factors have been suggested to be related with the presence of symptoms, such as the subtype, the parasite load, or factors associated to the host [7,8,9].

On the one hand, most gastrointestinal symptoms described in *Blastocystis* sp. carriers are similar to those attributed to irritable bowel syndrome (IBS), a functional gastrointestinal disorder characterized by abdominal pain, discomfort with defecation, and changes in the frequency or form of stool [10]. On the other hand, although the cause of IBS remains unknown, alterations in the gut microbiota including bacteria, parasites, fungi and even viruses have been suggested to be involved. Remarkably, post-infectious gastroenteritis (particularly parasitic infections) has been acknowledged as one of the strongest risk factors for developing IBS [11,12]. There are studies that have suggested a possible link between *Blastocystis* sp. infection and IBS, while other studies did not observe any association [13,14,15]. The aim of the present study is to assess the occurrence of *Blastocystis* sp. among asymptomatic subjects and patients with IBS in order to evaluate the potential association between IBS and the parasitic infection.

## 2. Methods

This is a cross-sectional study performed at Vall d’Hebron University Hospital (Barcelona, Spain) from April 2018 to December 2019. The asymptomatic group (healthy controls) inclusion criteria were: voluntary adult participants (18 years old or above) without gastrointestinal symptoms assessed through a structured and validated questionnaire (the Gastrointestinal Symptom Rating Scale, GSRS) [16]. The symptomatic group inclusion criteria were as follows: adult patients (18 years old or above) with IBS diagnosed according to Rome IV criteria [17]. Exclusion criteria for all participants were: any immunosuppressive condition, having been born outside Spain, having received any drug with demonstrated activity against *Blastocystis* sp. in the last 6 months before study inclusion (metronidazole, secnidazole, nitazoxanide, cotrimoxazole, paromomycin, iodoquinol, albendazole, and ivermectin), and the presence of cutaneous symptoms compatible with spontaneous chronic urticaria.

Demographic and clinical information was collected from all recruited participants. Moreover, the following epidemiological information was collected through a structured questionnaire: area of residence (urban or rural), regular contact with domestic animals, travelling abroad in the last 12 months or working in close contact with people (e.g., healthcare professionals, teachers, restaurant workers, caregivers). Two stool samples from the same defecation were obtained from all participants: one sample for the microscopic examination and one sample for *Blastocystis* sp. DNA detection by means of a Polymerase-Chain-Reaction (PCR) performed at the Parasitology Reference and Research Laboratory, National Centre for Microbiology (Instituto de Salud Carlos III, Madrid, Spain). Stool samples for *Blastocystis* sp. DNA detection were refrigerated at 4 °C until sent to the National Centre for Microbiology (the samples were stored up to two months). *Blastocystis* sp. infection was defined by the positivity of any of the diagnostic techniques. Information regarding the presence of other intestinal parasites was also collected.

### 2.1. Statistical and Ethical Issues

To calculate the sample size a prevalence of 10% was estimated among asymptomatic participants and 35% among symptomatic patients based on previous studies [18]. Assuming 5% of missing data, 41 subjects per group would provide >80% power to show significant differences between the two groups (at alpha < 0.05). Categorical data are presented as absolute numbers and proportions, and continuous variables are expressed as medians and interquartile ranges (IQR). The χ^2^ test or Fisher exact test, when appropriate, was used to compare the distribution of categorical variables, and the Mann-Whitney U test for continuous variables. Results were considered statistically significant if the 2-tailed *p* value was <0.05. SPSS software for Windows (Version 19.0; SPSS Inc, Chicago, IL, USA) was used for statistical analyses.

Procedures were performed in accordance with the ethical standards laid down in the Declaration of Helsinki as revised in 2013, and the study protocol was approved by the Ethical Review Board of the Vall d’Hebron University Hospital (reference number PR (AG) 29/2018). Written informed consent was obtained from all participants.

### 2.2. Microbiological Techniques

During the study period, the diagnosis of intestinal parasites, including *Blastocystis* sp., was performed by microscopic examination of a fixed and concentrated stool sample using a commercial device (Midi Parasep SF. APACOR. Wokingham, UK). Examination under the microscope was performed on the same day of collection. A sample of approximately 2 g of stool (size of a pea) is included in the Midi Parasep that is centrifuged at 1500 rpm for 3 min to decrease the risk of lysis of intestinal protozoa. A mix with methiolate and iodine was used for staining the samples before their examination. Parasite burden was classified as follows: low (<1 parasite/high-power field x400), medium (1–5 parasites/high-power field ×400), and high (>5 parasites/ high-power field ×400).

For molecular detection, DNA was extracted from 200 mg faecal samples using the QIAmp Fast DNA Stool Mini Kit (QIAGEN, Hilden, Germany). The extracted DNA was stored at 4 °C until analyses. Identification of *Blastocystis* sp. was achieved by a direct PCR protocol targeting the small subunit ribosomal RNA (*ssu* rRNA) gene of the parasite as described elsewhere (18). The assay uses the pan-*Blastocystis*, barcode primer pair RD5 (5′–GAGCTTTTTAACTGCAACAACG–3′) and BhRDr (5′–ATCTGGTTGATCCTGCCAGT–3′) to amplify a PCR product of ~600 bp. Amplification reactions (25 μL) included 5 μL of template DNA and 0.5 μM of each primer, 2.5 units of MyTAQ™ DNA polymerase (Bioline GmbH, Luckenwalde, Germany), and 5× MyTAQ™ Reaction Buffer containing 5 mM dNTPs and 15 mM MgCl_2_. Amplification conditions consisted of one step of 95 °C for 3 min, followed by 30 cycles of 1 min each at 94, 59, and 72 °C, with an additional 2 min final extension at 72 °C. Negative and positive controls were included in every PCR run. Obtained amplicons were separated by electrophoresis on 2% D5 agarose gels stained with Pronasafe (Conda, Madrid, Spain).

For sequence analysis, amplicons of the expected size were directly sequenced in both directions using the RD5/BhRDr primer pair in 10 μL reactions. DNA sequencing was conducted by capillary electrophoresis using the BigDye^®^ Terminator chemistry (Applied Biosystems Foster City, CA, USA) on an ABI PRISM 3130 automated DNA sequencer. Raw sequences were examined with Chromas Lite version 2.1 software (http://chromaslite.software.informer.com/2.1, accessed on 1 February 2021) to generate consensus sequences. These sequences were compared with reference sequences deposited at the National Centre for Biotechnology Information (NCBI) using the BLAST tool (http://blast.ncbi.nlm.nih.gov/Blast.cgi, accessed on 1 February 2021) to confirm that they belong to *Blastocystis*. Subtype and allele calling was carried out at the *Blastocystis* 18S database (http://pubmlst.org/blastocystis/, accessed on 1 February 2021). Blastocystis sequences generated in the present study were deposited in the GenBank public repository database under accession numbers MT913015 and MT913016.

## 3. Results

**Figure 1:**
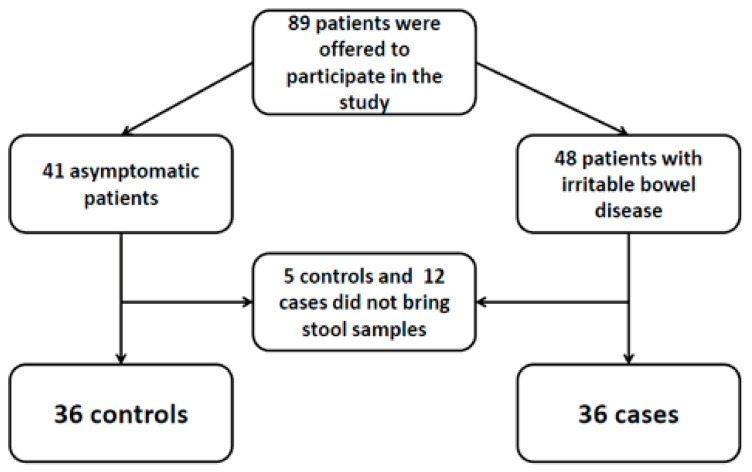
Flow diagram of patients.

Overall, 89 subjects were offered to participate in the study (41 healthy controls and 48 patients diagnosed of IBS-diarrhea). Five controls and 12 cases did not provide the stool samples and were excluded from the study; therefore, 36 controls and 36 cases were included in the analysis (see Figure 1). Thirty-five out of 72 (48.6%) were men and median age of participants was 34 (IQR 29–49) years. Regarding epidemiological factors, 4 (5.6%) participants were living in a rural environment, 32 (44.4%) had regular contact with animals, 30 (41.7%) were working in close contact with people, and 34 (47.2%) had travelled during the last year. Comparison of both groups regarding epidemiological factors is summarized in Table 1.

The overall occurrence of *Blastocystis* sp. detection was 27.8% (20/72). The positive rate assessed through microscopic examination was 22.2% (16/72), while the positive rate measured by PCR was 15.3% (11/72). Regarding the parasite burden among subjects with positive microscopic examination, in 6 (37.5%) patients it was low, in 6 (37.5%) patients it was medium, and in 4 (25%) it was high. Detection of concomitant intestinal microbial eukaryotes was only observed in four (5.6%) participants: two cases of *Endolimax nana*, one case of *Dientamoeba fragilis*, and one case of *Entamoeba hartmanni*. When comparing the occurrence of *Blastocystis* sp. detection between the two groups (irrespectively of the diagnostic technique performed), we did not find any statistically significant difference (see Table 2).

The *Blastocystis* sp. subtypes and alleles could only be determined by Sanger sequencing in two subjects, both of them belonging to the asymptomatic group: a 29-year old woman with high parasitic burden in the microscopic examination (ST1, allele 4), and a 54-year old woman with negative microscopic examination (ST6, allele 122).

## 4. Discussion

Here we compare the results of 36 asymptomatic healthy controls and 36 patients with IBS through microscopic examination of stool samples and PCR in order to detect *Blastocystis* sp. We did not find any statistically significant difference between both groups regarding the occurrence of *Blastocystis* sp. detection irrespectively of the diagnostic technique performed. Taking into account both detection methods (microscopic examination and PCR), the frequency of *Blastocystis* sp. identification among our study population was 27.8%. This rate is similar to those observed in other Spanish studies performed among adult patients, ranging from 17.3% to 35.2% [9,19,20]. However, Spanish studies performed among children showed prevalence slightly lower than in adults, ranging from 13% to 15% [21,22,23]. Age-related patterns for *Blastocystis* sp. carriage have been previously demonstrated both in developed and developing countries [24,25,26].

Surprisingly, the percentage of subjects with a positive *Blastocystis*-positive result by PCR was lower (15.3%) than that observed by microscopic examination (22.2%). PCR techniques for diagnosis of intestinal parasitic infections are usually more sensitive than classical parasitological techniques, although the last ones are highly observer-dependant, and sensitivity can vary widely from eye to eye [27]. Moreover, it is important to note that the great majority of positive cases by microscopic examination had a low or medium parasite burden (75%), which could explain the low percentage of positive cases detected by PCR. Suboptimal preservation of fecal samples and insufficient removal of PCR inhibitors during DNA extraction and purification may also account for the failure of PCR amplification reactions. The difference in the amount of stool sample analyzed must be also taken into account (2 g for microscopy versus 200 mg for PCR).

After decades of research, the pathogenic potential of *Blastocystis* sp. is not well understood [5]. The fact that the gastrointestinal symptoms in patients presenting with *Blastocystis* sp. in feces are very unspecific; the high percentage of asymptomatic carriers, and the coexistence of other possible causes of the symptoms (e.g., other viral, bacterial or parasitic agents), makes it very difficult to attribute the clinical symptoms to the presence of *Blastocystis* sp. Regarding IBS, contradictory results have been observed. One recent example is the one described by Shafiei et al., where 100 patients with IBS and 100 asymptomatic subjects were studied, with higher percentage of *Blastocystis* sp. carriage among IBS patients than in asymptomatic controls (15% vs. 6%, respectively) [28]. In contrast, Krogsgaard et al. described a higher proportion of *Blastocystis* sp. detection among control subjects (22%) compared to the one found in IBS patients (15%) [15]. A recent systematic review and meta-analysis that explored the role of *Blastocystis* sp. and *Dientamoeba fragilis* in IBS showed that individuals with *Blastocystis* sp. carriage were found to have a positive association with IBS (OR, 2.19; 95% CI, 1.54–3.13), while this association was not observed for *D. fragilis* carriage (OR, 1.13; 95% CI, 0.22–5.72) [29].

In our study population, percentage of *Blastocystis* sp. detection was higher in asymptomatic healthy controls than in IBS patients, although the difference was not statistically significant. A study performed in Malian children showed that *Blastocystis*-colonized children had higher gut microbiota diversity than *Blastocystis*-noncolonized children [30]. Indeed, some authors have regarded *Blastocystis* sp. as a constituent of the healthy gut microbiota [31]. High gut microbiota diversity is usually considered a healthy gut sign, since it decreases the possibility of having potentially pathogenic bacteria in the human gut. This fact could explain the results of the studies that show higher proportion of *Blastocystis* sp. carriage among healthy controls.

Another aspect that has been related to IBS is the *Blastocystis* subtype. A study performed among senior high school students in Indonesia observed an association between *Blastocystis* ST1 and irritable bowel syndrome-diarrhea [32]. Moreover, in the previously mentioned systematic review, considering the subtypes, meta-analysis results showed significant positive association for ST1 (OR, 4.40; 95% CI, 2.81–6.90) and ST3 (OR, 1.94; 95% CI, 1.36–2.77) to be potential risk factors for IBS [29]. Unfortunately, sequencing for ST detection could only be achieved in two participants of our cohort.

It has been suggested that parasite burden could be associated with the presence of clinical symptoms in patients with *Blastocystis* sp. detection, especially in those having >5 parasites/high-power field (×400) [2]. In our study, we did not observe any difference regarding the parasite burden between the two groups. This finding is in agreement with the results of other studies, such as the ones observed in the study published by Vargas-Sánchez et al., where 50 asymptomatic and 50 IBS patients with *Blastocystis* sp. detection were evaluated, and similar parasitological loads between the two groups were observed measured by quantitative PCR [33].

It is important to note that, when comparing epidemiological data between both groups in our study, the healthy group control had a higher proportion of subjects having travelled abroad in the last year before inclusion compared with the IBS patients group (72.2% versus 22.2% respectively, *p* < 0.001). A statistically significant difference remains when we only take into account travels to low- or middle-income countries. Recent travel has previously been described as a risk factor for *Blastocystis* sp. carriage, and could be a confounder when interpreting the results [9].

We acknowledge some limitations for our study. Firstly, as it is a cross-sectional study, we only have evaluated the stool samples from participants at one single time; it is well known that the gut microbiota changes over time. Another limitation of the study is that STs and alleles of positive cases could not be sequenced in most of the participants; hence the presence of IBS could not be related to specific STs (see more information in Appendix A).

## 5. Conclusions

In conclusion, the overall occurrence of *Blastocystis* sp. detection in our study was 27.8%, but no statistically significant differences were found between asymptomatic healthy controls and patients with IBS irrespective of the diagnostic technique performed (microscopic examination, PCR or both). Further studies are needed in order to establish the pathogenic potential of *Blastocystis* sp.

## Figures and Tables

**Table 1:** Comparison of epidemiological characteristics between healthy controls and patients with IBS.

	Healthy Controls (n = 36)	IBS patients (n = 36)	*p* Value
Age, years	30 (29–38.7)	43 (29.5–50.5)	0.094
Gender, male	19 (52.8%)	16 (44.4%)	0.479
Living in rural environment	3 (8.3%)	1 (2.8%)	0.614
Regular contact with animals	11 (30.6%)	21 (58.3%)	0.018
Travelling abroad in the last 12 months	26 (72.2%)	8 (22.2%)	<0.001
Travelling to low or middle income country in the last 12 months	14 (38.8%)	1 (2.7%)	<0.001
Working in close contact with people	20 (55.6%)	10 (27.8%)	0.017

**Note.** Data are reported as number (%) of patients or median (IQR).

**Table 2:** Microbiological results among healthy controls and patients with IBS.

	Healthy Controls (n = 36)	IBS Patients (n = 36)	*p* Value
*Blastocystis* sp. detection (microscopy + PCR)	13 (36.1%)	7 (19.4%)	0.114
*Blastocystis* sp. detection by microscopic examination	10 (27.8%)	6 (16.7%)	0.257
Parasite burden (n = 16) *			
Low (<1 parasite/high-power field ×400)	4/10 (40%)	2/6 (33.3%)	1
Medium (1–5 parasite/high-power field ×400)	4/10 (40%)	2/6 (33.3%)	1
High (>5 parasite/high-power field ×400)	2 (20%)	2/6 (33.3%)	0.604
Positive *Blastocystis* sp. PCR	8 (22.2%)	3 (8.3%)	0.101
Detection of other intestinal microbial eukaryotes	1 (2.8%)	3 (8.3%)	0.614

**Note.** Data are reported as number (%) of patients. * Among patients with positive microscopic examination.

## Data Availability

All data generated or analysed during this study are included in this published article.

## Appendix A

During the peer-revision process some concerns regarding the PCR technique raised. Inhibition controls are normally used in real-time PCR protocols. In our case, we took advantage of a robust, widely-used PCR protocol for the specific amplification of the ssu rRNA gene of *Blastocystis* sp. using pan-*Blastocystis* primers. Although we cannot completely ruled out that some of our false-negative results by ssu-PCR were the consequence of inhibition of the amplification reaction, we believe that the main problem was the long storage period (up to 2 months at 4 C) of the faecal material, that very likely has compromised the quality of the purified DNA and the diagnostic performance of the PCR. This point has been conveniently explained in the discussion section of the manuscript as a limitation of the study. Most of the *ssu*-PCR amplicons of the expected size generated faint bands on gel electrophoresis. In Sanger sequencing these bands generated poor-quality sequences that, although belonging to *Blastocystis* sp., did not allow the assignment to a specific subtype or allele calling. Therefore, we can confirm that those bands did not correspond to unspecific amplification reactions.
